# Feasibility Review of Aerated Materials Application in 3D Concrete Printing

**DOI:** 10.3390/ma16176032

**Published:** 2023-09-02

**Authors:** Magdalena Rudziewicz, Marcin Maroszek, Mateusz Góra, Paweł Dziura, Katarzyna Mróz, Izabela Hager, Marek Hebda

**Affiliations:** 1 Faculty of Materials Engineering and Physics, Cracow University of Technology, Warszawska 24, 31-155 Cracow, Poland; magdalena.rudziewicz@doktorant.pk.edu.pl (M.R.); marcin.maroszek@doktorant.pk.edu.pl (M.M.); mateusz.gora@doktorant.pk.edu.pl (M.G.); pawel.dziura@doktorant.pk.edu.pl (P.D.); 2 Faculty of Civil Engineering, Cracow University of Technology, Warszawska 24, 31-155 Cracow, Poland; katarzyna.mroz@pk.edu.pl (K.M.); izabela.hager@pk.edu.pl (I.H.)

**Keywords:** 3D concrete printing, sustainability, foamed concrete, thermal insulation, acoustic insulation

## Abstract

Recent years have witnessed a growing global interest in 3D concrete printing technology due to its economic and scientific advantages. The application of foamed concrete, renowned for its exceptional thermal and acoustic insulation properties, not only holds economic attractiveness but also aligns seamlessly with the principles of sustainable development. This study explores various solutions related to 3D printing technology in construction, discussing the design, production, and properties of foamed concrete mixtures. The integration of 3D printing and the potential for automating the entire process offers opportunities to boost productivity and reduce construction costs. Furthermore, the utilization of foamed concrete with its commendable insulation properties will enable a reduction in the usage of materials other than concrete (e.g., mineral wool, facade mesh, and polystyrene), significantly facilitating the recycling process during building demolition. This, in turn, will lead to the preservation of nonrenewable natural resources and a decrease in CO_2_ emissions. Despite the promising results, there have been limited studies focusing on 3D printing with foamed materials, whereas a survey of the existing body of literature indicates a notable absence of endeavors pertaining to the utilization of aerated concrete within the realm of 3D printing, especially geopolymer composites (GP) and hybrid geopolymer composites (HGP). The outcomes delineated in the ensuing discourse are demonstrative for conventionally used materials rather than the additive manufacturing variant. Hence, this work aims to systematically review existing practices and techniques related to producing foamed concrete with 3D printing technology. This analysis also contributes to the establishment of a foundational framework and furnishes a preliminary basis upon which future endeavors aimed at the 3D printing of aerated concrete can be embarked. The findings from the literature analysis justify the desirability of continuing research on this topic, particularly when considering the potential for large-scale industrial implementation. This article provides a comprehensive state of the knowledge on the development of 3D printing techniques for foamed concrete mixtures. By consolidating and analyzing findings from different studies, this article offers insights into the advancements, challenges, and potential applications of foamed concrete in additive manufacturing processes. This, in turn, contributes to the overall understanding and advancement of 3D printing technologies using foamed concrete as a versatile and sustainable construction material. The encouraging results obtained from the analysis further underscore the need for the continued exploration of 3D printing, especially with an eye towards its industrial-scale implementation.

## 1. Introduction

Expanded concrete (cellular concrete) is a lightweight material that is obtained by introducing and enclosing gas inside a concrete structure. Foam concrete is one of the varieties of expanded concrete, and its name comes from the method of production. A foaming agent is added to the cement mixture, as a result of which, at ambient temperatures, a material with a porous structure is formed, in which the pore volume is at least 20% [1]. Another example of expanded concrete is aerated concrete, which is produced by chemical reactions between the blowing agent and the concrete. The most used aerated agent is aluminum powder [2,3,4]. Aerated concrete hardens at high temperatures and high humidity. Both foam concrete and aerated concrete are considered environmentally friendly materials.

The universality and very good thermal insulation properties of cellular concrete affect its widespread use in low-energy construction, which is part of the sustainable development trend [5]. Cellular concrete is most often produced in the form of blocks, which do not require additional insulation during the building of walls. Moreover, depending on the needs and requirements of the structure, the density of the designed foamed materials can be freely controlled in the range from 200 to 1900 kg/m^3^ [6]. However, concretes with a density of less than 400 kg/m^3^ are considered suitable materials for structural applications, but rather, they are used as insulation material [7].

### 1.1. Expanded Concrete 

As research and technology advancements continue, expanded concrete has the potential to play a significant role in the future of construction, enabling the creation of more sustainable, cost-effective, and energy-efficient buildings and infrastructure. From a technical and scientific perspective, these key characteristics contribute to its potential application in emerging technology that is additive manufacturing. Expanded concrete’s unique combination of properties, including reduced density, enhanced thermal insulation, good flowability, high compressive strength, fire resistance, and versatility, positions it as a promising material for large-scale 3D printing. The integration of 3D printing and the potential for automating the entire technological process present an excellent opportunity to enhance productivity and concurrently decrease construction costs. Furthermore, the additive technology may enable control of the geometry of the manufactured structure both globally as the entire body of the building and locally on the cross-section of the wall, enabling the production of solid and foamed areas or with designed air pores. In addition, during the manufacturing process, it is possible to change the composition and thus the properties of the material used; this feature will help facilitate both the structural and insulation requirements. 

### 1.2. Additive Manufacturing Technology

Three-dimensional printing technology allows the creation of buildings or their elements with complex geometry, which previously would not have been profitable due to the need to use conventional methods. Many concrete structures have been built in 3D printing technology, e.g., a two-story building in Dubai by Apis Cor [8], a communal village in Austin by ICON [9], concrete vehicle arches in California by US Marines Corps [10], COBOD and Peri Group homes in Berlin [11], a river revetment in Suzhou by Winsun [12] and pedestrian arch bridge in Shanghai [13], and five residential houses of the Project Milestones in Eindhoven, the Netherlands [14]. Despite such a wide range of construction projects, the use of 3D concrete printing for standard structures is rare due to technical challenges and legal restrictions. Standard concrete mixes cannot be used in the 3D printing process due to the technological limitations of the process, and above all, the noncompliance of the rheological properties of the conventional material with the requirements of the printing process. Therefore, it is extremely important to study various mixtures and the ways of their applications in the future to facilitate the development of an alternative, ecological way to build private and public buildings.

Buildings erected with the use of 3D printing technology do not need to use additional external insulation, plasterboards, or reinforcement meshes, which not only increase construction costs but also hinder the possible recycling of such a building, as only “clean” concrete waste can be reused. This will save deposits of nonrenewable natural resources. In contrast to conventional construction methods, the utilization of foamed concrete in 3D printing technology allows for the elimination of additional external insulation, gypsum board, or reinforcement meshes. This not only leads to cost reductions in construction but also facilitates the recycling process, as waste from foamed concrete buildings can be reused. The recycling of construction waste plays a pivotal role in combating the escalating global waste issue. Nevertheless, the scientific literature suggests that contaminants from various materials used in construction, such as gypsum, wood, plastic, steel, or glass, can impede the efficiency of the recycling process. The multiplicity of materials generates a very high probability of contamination transferring to the new material. They can reduce the compressive strength of concrete and reduce the modulus of elasticity by 15% compared to the reference sample [15].

The exclusive use of foamed concrete for building houses is expected to contribute to the advancement of the construction materials recycling industry. The absence of a need to segregate different materials enhances the efficiency of the recycling process and allows for the greater utilization of waste in the production of new concrete. Additionally, the growth of the construction sector employing additive manufacturing (3D printing) may serve as a market for materials derived from the recycling of construction waste. In the context of environmental conservation and the reduction of waste accumulation in landfills, the sustainable management of construction waste holds the utmost importance. According to the European Commission (europa.eu) [16], construction and demolition waste (CDW) accounts for over one-third of all waste generated in the EU [16,17]. For example, in 2016, 2.5 billion tons of waste was produced in Europe, of which 36.4% was construction waste. Although a portion of construction waste is recycled (e.g., cleaned rubble into ground aggregate can be used, for example, to harden the ground, produce asphalt or road surfaces, and for the production of foundations), a large part of construction waste is still landfilled [18,19,20]. Therefore, more and more entities are involved in research on the development of appropriate concrete or geopolymer mixtures containing recycled raw material and their use in large-scale production as additives [21,22,23]. Until now, Portland cement concrete, composite or sulfoaluminate, and sand or recycled aggregate have been used as aggregates [24,25,26,27,28]. However, data on mixes and printing parameters are most often the results of implemented projects and constitute the know-how of the companies preparing them. Thus far, few studies have addressed the possibility of 3D printing with foamed materials. Therefore, this work aims to review practices, systematize knowledge, and learn techniques related to the possibility of producing foamed concrete with 3D printing technology.

## 2. Additive Manufacturing with Foamed Concrete

**Figure 1:**
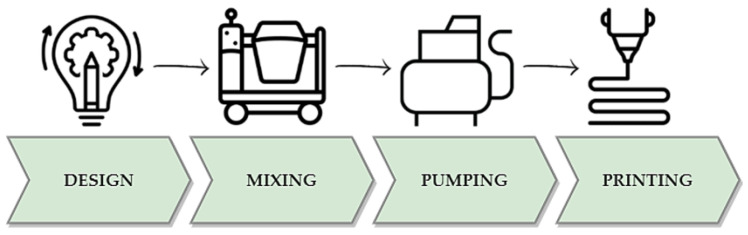
Steps in additive manufacturing with foamed concrete.

The disparities between conventional concrete designed for 3D printing and foamed concrete necessitate the development of dedicated printing processes and material transport systems, particularly for large-scale applications. Given these distinct material compositions, specialized 3D printing processes and material transport systems are essential for each concrete type. The successful large-scale implementation of 3D printing with foamed concrete relies on addressing several key challenges unique to this material. The foam content and its distribution within the concrete mix shall be meticulously controlled to achieve the desired mechanical properties and printability. As a result, it becomes imperative to customize the material transport system to effectively handle the unique flow properties of the foamed concrete mix, ensuring a seamless and consistent supply to the printhead, which is located on either the robot’s arm or the gantry structure [29]. This advanced printing process represents an innovative construction approach, enabling complex geometries and structures to be created with high precision and efficiency. The first critical step in concrete printing involves the meticulous preparation of the proper concrete mix, which is a key factor in ensuring the success and quality of 3D-printed structures. Subsequently, the mixing process takes place, involving the incorporation of stable foam into the cementitious slurry and the creation of a cellular structure with air voids. Proper mixing uniformly distributes the foam throughout the concrete mix, enabling a consistent distribution of air voids within the material. This, in turn, imparts the characteristic lightweight nature of foamed concrete, which is crucial for achieving a reduced material weight and enhanced thermal and acoustic insulation properties. Next, the paste is transported to the printhead to precisely place the material on the working field layer by layer. Figure 1 depicts the overall 3D printing setup. The material formed in this way sets at a properly selected time, depending on the mixture. The bonds between the layers are created as a result of the pressure exerted by the next new band applied by the device [30,31].

The basic parameter used to assess the prepared mixture for the possibility of its use in 3D printing includes the ease of pumping continuously without blocking the material inside the nozzle. Therefore, the material must be fluid enough for its transport to run smoothly, but on the other hand, too much fluidity may result in the uncontrolled spilling of the mix out of the nozzle [32]. This is assessed with the buildability parameter, which also considers the aspect of a deformation analysis of the printed layers. It is crucial to bear in mind that the tensile stress induced by nozzle displacement during the extrusion process results in the elongation of pores within the printed sample [33]. The possibility of building subsequent layers depends on the yield strength of fresh concrete, which is divided into static yield strength (important when the nozzle begins to shape the material) and dynamic yield strength (vital in the further stages of building layers) [34,35]. It is very important to eliminate the possibility of the structure collapsing under its own weight [30,36,37]. Most often, the cause of destabilization of the structure is random displacements and the loss of equilibrium of forces [38]. 

## 3. Modern Foamed Concrete

A significant reduction in the self-weight of the structure can be achieved by using foamed concrete with a lower density (200–1900 kg/m^3^) than conventional concrete (2400 kg/m^3^), which is of great importance in the context of building with the use of 3D printing technology [39,40]. Thanks to the decreased density, the thermal insulation properties of the foamed material are higher than those of traditional materials [41]. The lower density and air voids created by the stable foam contribute to enhanced thermal insulation, making foamed concrete an efficient choice for reducing heat transfer and improving energy efficiency in buildings. As a result, the use of foamed concrete can contribute to reducing the thickness of standard thermal insulation in the building envelope. Moreover, foamed concrete exhibits a high compressive strength, ensuring its ability to withstand considerable loads and structural demands. This property is essential for constructing robust and durable buildings and infrastructure. Foamed concrete also possesses satisfactory acoustic insulation properties [42], making it an effective material for reducing sound transmission and enhancing acoustic comfort within buildings. Another notable advantage of foamed concrete is its fire resistance of class A1, thanks to its inorganic composition [43]. The stable foam in the concrete mix acts as a natural fire barrier, providing added safety and protection against the spread of flames. According to the tests carried out by [44], to achieve the required fire resistance for structural application, the density of foam concrete should exceed 250 kg/m^3^. In addition to its thermal, mechanical, and acoustic properties, foamed concrete exhibits a resistance to moisture and fungi, making it a suitable material for applications in humid or damp environments. Its low water absorption capacity further enhances its durability and resilience to environmental factors [45]. The easy processability of foamed concrete allows for flexible shaping and molding, making it a versatile material for creating complex geometries and customized designs in construction projects [36,46]. Therefore, it may be considered an environmentally friendly material with a clear economic potential [47]. Above the promising properties, the production of foamed concrete confirms its environmental friendliness. Its production often uses recycled raw materials (fly ash, pre-ground foam concrete, sand, granite slabs, chalk, and glass) [48,49]. Also, there are ongoing numerous research projects on incorporating construction demolition waste (CDW) into production technology. Despite the many advantages, foamed concrete is not yet widely used for additive manufacturing due to the challenges of the manufacturing process of this material for printing applications [50].

### 3.1. Techniques for Producing Foamed Concrete Materials and Their Suitability for 3D Printing

**Figure 2:**
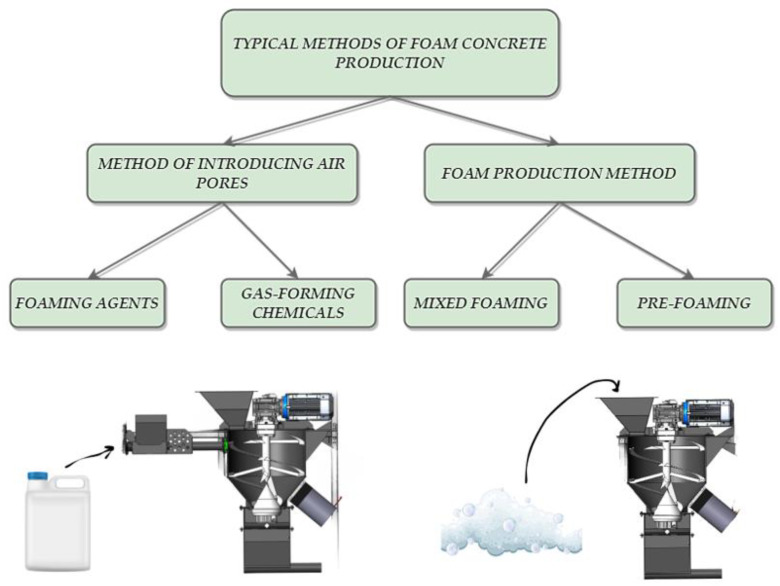
Production process of foamed materials with foaming agents and pre-foaming.

The production of foamed concrete materials involves various methods, each of which plays a crucial role in determining the final product’s properties. A key objective in this process is to introduce technical foam into the cement mix, creating air pores within the structure while carefully selecting an appropriate foaming agent. The overall production of foamed concrete can be categorized based on the method of generating air pores in the material: chemical methods, involving the use of substances like aluminum powder, silica fume, or hydrogen peroxide that release gas through a reaction, or the use of foaming agents [46]. In the realm of foam concrete production, two fundamental methodologies hold prominence: mixed foaming (intensive mixing) and pre-foaming (where the concrete mix and foam are prepared separately). In the mixed foaming approach, the prepared material undergoes mixing with a surfactant, yielding a foam that imparts the distinctive porous structure characteristic of aerated concrete. Conversely, the pre-foam technique allows for more refined control over the density of the final mixture by incorporating a specific amount of foam into the prepared material. This technique finds extensive usage within the construction industry, with the resultant material serving as a foundational element for floors, roads, and parking lots and functioning as a thermal and acoustic insulation layer [51,52]. Figure 2 presents an overview of the diverse foamed concrete production methods. Both chemical and foaming agents are deployable in either of these approaches. However, a precise definition of the composition and quantity of foam becomes critically imperative in the context of the pre-foaming method, as it directly influences the achieved outcomes. The intricacies and efficacy of these production techniques continually captivate researchers and practitioners, with persistent endeavors aimed at refining foam concrete’s performance across a spectrum of construction applications. Indeed, the continued advancements in foam concrete production methodologies (including additive manufacturing) signify a profound contribution to the burgeoning use of this versatile material within sustainable and pioneering construction practices.

The use of blowing agents has great potential for 3D printing, mainly due to the ability to control the properties of the mixture. The final volumetric shape of air pores in the material structure depends on the quality of the foaming agent and the technology of its application. The greater the content of air pores, the higher the plastic viscosity (the ability to flow under the applied shear stress) [53]. The higher the volumetric content of the air, the lower the gravitational force associated with it. The number of pores also affects the density and strength of the finished material; as the foam itself is thermodynamically unstable and its disintegration occurs over time, it is important to select agents of appropriate quality [54,55]. For economic reasons, synthetic foaming agents are most often used for this purpose. They are cheaper than those based on natural raw materials, can be stored for a long time, and, additionally, are less susceptible to significant temperature amplitudes. These agents can be used in both conventional foamed concrete manufacturing techniques described. Nevertheless, to ensure the easy pumping of concrete for 3D printing, air pores should be smaller than 1 mm. Compact pores are less susceptible to damage or compression during material pumping. As per the findings of the research [56], internal pressure exerts an influence on the air pores. Diminished internal pressure renders larger pores more susceptible to deformation, whereas smaller air pore diameters correspond to elevated internal pressures within the bubbles. Other researchers have demonstrated that the pores with a geometry ranging from 0 to 1 mm^3^ displayed an almost spherical morphology, with each pore maintaining its independence from adjacent pores. Furthermore, these pores were distributed in a relatively uniform manner [57]. Furthermore, with an increase in foam volume, the merging of bubbles leads to the emergence of a broader range of void sizes, ultimately resulting in diminished strength [48]. According to the literature analysis, synthetic foaming agents generate much larger air pores compared to agents based on natural raw materials (proteins) [58]. The larger size of the pores formed in the material negatively affects the strength of the concrete and the number of pores [59]. Most of the studies presented in the literature are based on the use of proprietary foaming agents for testing [60,61,62]. However, the research is focused on determining the parameters of the concrete mix to produce foamed concrete with a given density and properties. Simultaneously, uniform sample density does not imply an even distribution of air voids within them, whereas the arrangement of pores significantly influences the compressive strength of foamed concrete, and larger air voids inside the sample result in reduced compressive strength [63]. The exact requirements that must be met for 3D printing with foam concrete have not been defined so far.

Undoubtedly, the key processes in additive manufacturing are mixing and pumping the produced composition into the printhead. Despite the requirements, according to the literature data, devices available on the market can be used to integrate materials [64] inter alia:

(a) A planetary mixer has arms that move called planetary movements, and the mixing trajectory is continuous. The mixing blades are set at such an angle that the mixing efficiency is as high as possible. The resulting material, together with the hardener, is pumped to the foam generator, where the ingredients combine and foam is produced. The pumping takes place continuously, and the resulting foam is transported through a flexible hose to the nozzle [65].
(b) A conical mixer is equipped with two counter-rotating mixers mounted coaxially in the center axis of the device; thanks to which, a homogeneous concrete mix is created. The screw agitator transports the material vertically upwards, thus setting it into rotation. The blades of the second agitator are set on the outside work in the opposite direction to this movement. Such intensive mixing guarantees the accurate distribution of all ingredients and obtains a homogeneous mass. The mixing of the foam with the concrete slurry must occur at lower speeds than the mixing of the concrete material itself; otherwise, the stability of the preformed foam is significantly reduced. In addition, the frequency of feeding the prepared foam to the concrete material is important, as providing all the foam at one time negatively affects the homogeneity of the final material [60].
(c) The cavitation disintegrator is a device that shreds the material in two ways: cavitation and impact. The principal operation of the device is to introduce high-intensity ultrasonic waves into the process medium, which alternately generates high- and low-pressure cycles during which cavitation bubbles are formed. After absorbing enough energy, they implode, and the resulting shear forces cause collisions between solid particles and breaks them into the expected size. The resulting material is homogeneous. The cavitation phenomenon occurs in the profiled grooves located near the impeller. To create zones of low and high pressure inside the tank, it is important to achieve the appropriate rotational speed [66,67].
(d) Turbulence mixer is the mixer equipped with a vertical shaft, and the tank is in the shape of a cone. The mixture that is produced in this device can be pumped under foam concrete pressure directly to the printing head [60].

The pump responsible for transporting the material to the printhead is not in the printing area but far beyond it. The pumping results depend on the parameters of the pump and the diameter of the hose, and the distance that the material must travel, as well as the selected pumping methodology. The pumped material should be stiff enough to not change its shape after building a layer but fluid enough for transport without disturbances. The potential accumulation of material inside the hose makes it necessary to replace it frequently, so it cannot be kept for a relatively long time. On the other hand, the short flexible hose limits the size of the printing field. The mixture should have a low viscosity and a moderate yield strength [68]. The pumps used in this process are most often piston, worm, and centrifugal pumps.

At the Dresden University of Technology, under the CONPrint3D Ultralight Project, research was carried out to produce foamed concrete suitable for 3D printing, and an attempt was made to develop a technique for feeding this material to the printhead. Several concepts were explored, such as (a) manual filling of the printhead reservoir, (b) mixing and pumping (the mixer was directly connected to the foam delivery pump), (c) pumping to the integrated mixing system (the mixer was connected directly to the printhead), and (d) the system where the mixer was placed on the printhead and the supply of raw materials was automated; an important advantage of this concept was the fact that the pumping process did not affect the stability of the produced material in any way [69]. Despite checking many possibilities of feeding the mixture, each of them turned out to have some limitations, such as the involvement of additional manpower, slow pace of work, restrictions on the size of the building due to the location of the pump outside the printing area, and the need to use a flexible hose or a technologically complex mixing system of ingredients immediately before extrusion. The authors showed that the durability of the printed layers of foamed concrete is influenced by both the rheological properties of the mix and the mixing parameters. In the experiment [41], it was proven that increasing the intensity of mixing improved the compressive strength by 70%.

### 3.2. Overview of the Studied Foamed Concrete Mixtures

During additive manufacturing with foamed concrete, the mixing process must be carried out in such a way that the disintegration of air pores is as small as possible. Considering the expected low density of the produced material and the low yield strength required for the process, the composition of the mixture is important, in which the required parameters, (e.g., density) will be achieved with a reduced content of technical foam. Scientists have so far studied numerous compositions, focusing on different properties. Inter alia, the research revealed that a blend comprising Portland cement, microsilica, superplasticizer, and fly ash was employed as the binding agent. The authors examined the structural integrity of the layered elements, assessing both the compressive and flexural strength characteristics [70]. Scientists studying geopolymer foam for 3D printing used Class F fly ash, ordinary Portland cement, silica fume, and fine lime powder. The activators used in this study included sodium silicate solution (water glass), sodium hydroxide (NaOH), and sodium sulfate (Na_2_SO_4_). Lightcrete 02™ liquid surfactant was used as the foaming agent and foam stabilizer. The investigation revealed that the thermal conductivity of the printed foamed matrices was comparable to that of various other insulation materials, including foamed concrete, expanded clay, cork boards, and porous fly ash-based geopolymers. The selected suspensions exhibited shear yield stress values ranging between 60 and 130 Pa. Remarkably, the diminished yield stress observed in the foamed suspensions did not impede their buildability due to their correspondingly reduced density. [71]. In a separate investigation, the researchers amalgamated Portland Type II cement with the inclusion of coal fly ash, SiO_2_, Al_2_O_3_, and a polycarboxylate ether superplasticizer. The introduction of a protein-based foaming agent facilitated the generation of the requisite foam composition. The examined component exhibited a thermal conductivity of 0.24 W/mK, signifying an improvement exceeding 80% in comparison to the reference material. Furthermore, the compressive and flexural strengths were evaluated at the 28-day mark, registering values of 10.40 MPa and 2.12 MPa, respectively [59]. The experimental analysis demonstrated that the incorporation of hydroxypropyl methylcellulose in foam concrete substantially enhances the pore dimensions, with the variations in pore orientation being contingent upon the dosage of the admixture [56]. Furthermore, the research revealed that the introduction of hydroxypropyl methylcellulose and silica fume leads to a heightened plastic viscosity and yield point, with a pronounced increase in the yield point observed primarily with elevated levels of silica fume [56,67]. Reducing the share of technical foam while increasing the mechanical properties of conventional concrete was achieved by replacing sand with (e.g., expanded perlite and pumice). The compressive strength of cubic specimens exhibited a rise of 75% and 180% at the 7-day mark and 65% and 188% at the 28-day interval [72]. While examining the behavior of foamed concrete with the addition of expanded perlite as the aggregate, five mixes with a density of 800–1355 kg/m^3^ were prepared. The yield strength and viscosity for these materials were higher than for sand-based materials [73]. Another study showed that aggregate from expanded glass beads as a substitute for sand allowed it to achieve a material density of 1015 kg/m^3^, but its strength decreased by 50% compared to the reference material [45]. Interestingly, replacing 30% of the sand with expanded clay improved the printability and had a positive effect on the modulus of elasticity and strength of the sample [74]. Moreover, a negligible amount of sand causes the shrinkage of concrete to decrease and the yield strength to increase [7]. There was also a tendency to increase the density with the increase in the number of layers during the printing of the walls. Almost all analyzed experiments were focused on testing foamed concrete with a density above 800 kg/m^3^. This is related to the dependence of the increase in the heterogeneity of the foams with the decrease in the density of the produced material. The effects of the inhomogeneity and delamination of the foam and base mix negatively affect the printing process [61].

The essence of the process of erecting a building using 3D concrete printing technology is the proper selection of the printing parameters, as well as a properly defined path along which the printing head moves. For example, the appropriate design of the tested sample with a truss cross-section allowed saving 46.7% of the material while ensuring 90% of the strength of the tested element. The material used in this study contained expanded clay sand [61]. Employing heterogeneous compositions of foamed concrete within a singular construction scenario, e.g., the use of hardened, printed mineral foam and fiber-reinforced concrete for the construction of some formwork elements, saves up to 72% of the total volume of concrete and as much as 70% of the weight of the tested element [75].

It has been shown that foam concrete mixtures may achieve good engineering properties. The foam concrete composition with a density of 1100–1580 kg/m^3^ in studies [56] was characterized by a low thermal conductivity of 0.24 W/mK, along with a simultaneous compressive strength exceeding 10 MPa. A high compressive strength value of 8.3 MPa was also achieved for a material with a density of 1200 kg/m^3^, in which 5% of the cement volume was replaced with aluminosilicate [61]. On the other hand, for the concrete made of fly ash with a density of 600–1000 kg/m^3^ and a porosity of 60–70%, it was found that a high proportion of surfactant negatively affects the shape of the printed layer [61].

The team from Technische Univeraität Dresden [61] carried out tests of various material mixtures using Portland cement, fly ash, silica fume, and aluminosilicate. During printing, the nozzle did not clog, but the surface quality of the layers was noticeably lower as the wall height increased. The time that passed from adding water to the mixture was detrimental to the surface of the printed layers. The printing time after mixing must therefore be short, which is important given the need to transport the mix to the construction site [61]. In the case of using a cavitation disintegrator, the results were not satisfactory enough (the obtained material density was 1412 kg/m^3^). In further research, different amounts of foaming agent and different mixtures were used, but the same mixers were used: a cavitation disintegrator and a turbulence mixer, and it was thanks to the latter that a lower foam concrete density was obtained even when a smaller amount of foaming agent was added. At the same time, the study of water absorption of the tested samples showed that the quality of the pores obtained using a turbulent mixer is better.

Moreover, analyzing the works of the authors, foam concrete suitable for printing showed low thermal conductivity and relatively high compressive strength above 10 MPa at a density of 980 kg/m^3^. The thermal conductivity of the tested mix of printable foam concrete is 0.24 W/mK, which is about 85% less than conventional fine-grained concrete (density 2100 kg/m^3^), which has a thermal conductivity of 1.68 W/mK (a value close to poured concrete) [59,76].

To obtain a quick setting in the process of printing with conventional concrete, types of cement with shorter setting times are usually selected or accelerating admixtures are used. These solutions can also be applied to foam concrete. However, the use of setting accelerating materials in foam concrete does not always give the same effect as in ordinary concrete [77]. Table 1 provides a concise overview of the aforementioned information.

## 4. Sociological and Ecological Considerations

An increasing problem in the sense of stability and residential opportunities for young people in Poland is the high prices of houses and flats, which results not only from the growing demand but also from the prices of materials and rising labor costs. The possibility of erecting buildings using 3D printing technology will contribute to reducing the demand for human labor, which is today a significant barrier in the construction industry due to rising salaries but, above all, the lack of qualified construction workers [70]. In 2022, the construction sector experienced an average of over 390,000 job vacancies each month, marking the highest level ever recorded while simultaneously maintaining a relatively low rate of unemployment within the industry [78]. Physical and hard work, which is undoubtedly work on a construction site, is no longer attractive to young people brought up in the era of digitization [79]. The use of additive manufacturing in this part of the economy is a big challenge, but at the same time, it will create jobs that will be interesting for the younger generation; what is more, such a scenario will affect the development of this slightly conservative branch of industry.

In addition, the construction industry is responsible for almost 50% of global carbon dioxide emissions and 20–50% of natural resource consumption, most of which is related to significant land exploration, which has a significant impact on the natural environment [80]. Therefore, the development of the construction industry at the global level is important for solving problems related to climate change, reduction of greenhouse gas emissions, and lowering energy consumption [81,82]. The use of ecological and energy-saving construction methods will contribute to saving water, energy, and fossil fuels and reducing greenhouse gas emissions. The development in the field of 3D printing with foamed concrete will allow the construction of buildings that are in line with the philosophy of the circular economy (CE), because they will not need an additional layer of insulation that prevents the subsequent recycling process of the erected walls. Meanwhile, the reuse of “clean” construction waste from the demolition of a 3D-printed house will allow for the cheaper and much more environmentally friendly construction of subsequent buildings. This will significantly reduce the impact of construction waste on the environment. It can therefore be assumed that the discussed revolution in the construction industry will significantly affect the sustainable consumption of resources and will allow for the development of the recycling of construction waste.

## 5. Conclusions

Due to the nature of additive manufacturing technology, concrete mixes intended for printing must meet specific requirements in terms of material properties. The growing interest in concrete 3D printing means that the developed mixes and the printing preparation technology must be repeatedly checked and evaluated very critically. So far, in the construction market, foam concrete has been used as structural material for low-rise buildings or a filler element in skeletal structures. Foamed concrete exhibits valuable characteristics, such as reduced density, enhanced thermal insulation, good flowability, high compressive strength, fire resistance, and versatility, making it an environmentally friendly material with potential applications in sustainable construction. Large-scale 3D printing using foamed concrete offers the advantage of eliminating the need for additional external insulation, plasterboards, or reinforcement meshes during construction, leading to cost reduction and simplifying the recycling process. The ability to control the geometry and composition of the manufactured structure allows for tailored designs that meet both the structural and insulation requirements. Despite the conducted research on this material, one method of preparing a foamed concrete mix for 3D printing has not yet been developed. First, it is necessary to determine the composition and proportions of the composition, which will make it possible to pump and build layers. Addressing challenges unique to foamed concrete, such as precise control of the foam content and distribution, is crucial for achieving the desired mechanical properties and printability. The integration of foamed concrete with 3D printing offers innovative opportunities for construction, enabling the creation of complex geometries with high precision and efficiency. There are two techniques for producing foamed compositions, depending on how the foam is produced and how pores are generated in the mix. Pre-foaming and mixed foaming are ways of introducing foam into a concrete slurry. Air pores may be caused by the addition of chemicals or blowing agents to the mixture. The use of advanced components of the concrete mix makes it possible to control its parameters, such as setting speed, mechanical strength, fire resistance, thermal insulation, and aesthetics. Most of the information available in the literature presents the results of experiments based on the empirical method of material composition design. The methods of foam concrete production in the analyzed research works were the use of foaming agents and pre-foaming. The number of air pores inside the material depends on the foaming agent used and the application technology. The ease of pumping foamed concrete is influenced by the pores formed, and their diameters should not be greater than 1 mm. The ability of foamed concrete to form layers during printing is considered ideal when the size and shape of the layer faithfully reflect the width of the nozzle. The density of the foamed concrete decreases during the curing and drying of the already printed elements. However, as the number of printed layers increases, there is a noticeable tendency to increase the density. Despite the promising results achieved so far by scientists studying foam concrete for 3D printing, it is necessary to conduct further research on this material and the printing process itself. Achieving the desired density and mechanical properties while maintaining printability is a key focus. Notably, the use of specific mixtures and additives has shown promise in achieving high compressive strength and low thermal conductivity, making foamed concrete a viable option for sustainable construction practices. The choice of foaming agent and production method significantly influences the properties of the foamed concrete material. Synthetic foaming agents are often favored for their economic benefits and stability. Mixing techniques, such as the use of a turbulent mixer, have demonstrated improved foam concrete density and quality of the pores. The development and implementation of additive manufacturing with foamed concrete material will undoubtedly contribute to the reduction of global carbon dioxide emissions, the consumption of natural resources, and the reduction of thermal energy consumption. Buildings without reinforcement meshes or additional external insulation are easier to demolish, and the “clean” construction waste will be able to be reused for the construction of other buildings.

Overall, additive manufacturing with foamed concrete holds significant potential for revolutionizing construction practices and facilitating the development of eco-friendly and efficient building structures. Further research and advancements in foamed concrete production and 3D printing technologies are crucial to unlock the full potential of this innovative construction material.

## Figures and Tables

**Table 1:** Catalog of the examined formulations and their pivotal properties specific to three-dimensional printing concrete.

Classification	The Main Components of the Ingredients	Crucial Properties of 3D Printing	Authors	Year
foam concrete	Portland cement, Isparta, Kayseri, Nevşehir, and Karaman pumices, Limestone powder, Al_2_O_3_, Fe_2_O_3_, Protein-based foam agent, Cellulose,	Yield strength, Viscosity, Compressive strengths, Thermal conductivity, Flexure strength	Ş. Kilincarslan et al. [73]	2018
3D-printed foam concrete	Portland cement, Micro-silica, Superplasticizer, Fly ash	Extrudability, Pumpability, Buildability, Flexural strength,	V. Mechtcherine, et al. [70]	2019
3D-printed foam concrete	Fly ash, ordinary Portland cement, Silica fume, Lime powder, Sodium silicate, Sodium hydroxide (NaOH), sodium sulfate (Na_2_SO_4_), Liquid surfactant	Extrudability, Shape retention, and buildability Thermal conductivity	H. Alghamdi and N. Neithalath [76]	2019
3D printable concrete	Cement, Microsilica, Fly ash, Fine quartz sand (Fraction 1: 0.06–0.2 mm), Natural river sand (Fraction 2: 0–1mm and Fraction 3: 0–2 mm), Polycarboxylate ether superplasticizer,	Extrudability, Stability	V.N. Nerella et al. [77]	2019
3D-printed foam concrete	Portland Type II cement, Coal fly ash, SiO_2_, Al_2_O_3_, Polycarboxylate, protein-based foaming agent	Thermal conductivity, Flexural strength, Compressive strength	V. Markin et al. [59]	2019
geopolymer foam concrete	Geopolymer binder: furnace slag with a partial substitution of fly ash, Sodium metasilicate powder Sand, Expanded perlite, Sodium dodecyl sulphate solution as the foaming agent, Polyvinyl alcohol (PVA) fiber (8 mm length)	Compressive strength, Thermal properties and performance	K. Pasupathy et al. [72]	2020
3D printable concrete	Portland cement, Fly ash, Quartz sands were used as coarse and fine aggregates, respectively, Polycarboxylic ether-based superplasticizer, Hydroxypropyl methylcellulose as a viscosity modifying agent, Polypropylene fibers (12 mm length)	Extrudability, Buildability, Desorptivity	A.V. Rahul and M. Santhanam [74]	2020
3D printable concrete	Rapid-hardening Portland cement, Silica fume, Limestone powder, Al_2_O_3_, Fe_2_O_3_, Polycarboxylate-based superplasticizer, Viscosity modifying admixture, Fine silica sand (surface area of 0.11 m2/g and an average size 0.25 mm)	Shear stress, Tensile strength, Sample weight	Li Wang et al. [75]	2020
3D-printed foam concrete	Portland cement, Coal fly ash, Pozzolanic additives of silica fume and alumosilicate, Polycarboxylate ether-based high-range water-reducing admixtures, Protein-based foaming agent	Foam stability, Homogeneity and workability, Compressive strength, Thermal insulation	V. Markin et al. [59]	2021
3D-printed foam concrete	Portland cement, Hydrophobic surface-modified expanded perlite, Fine sand, Polycarboxylate ether-based superplasticizer, Sodium dodecyl sulfate	Static yield stress, Dynamic yield stress, Plastic viscosity, Viscosity Recovery, Printability, Buildability, Compressive strength	K. Pasupathy et al. [45]	2022
3D-printed foam concrete	Ordinary Portland cement, Silica fume, Hydroxypropyl methylcellulose, Nanomodified compound foaming agent, River sand (three distinct grades of sand with maximum particle sizes of 0.6 mm, 1.18 mm, and 2.36 mm were utilized)	Rheological properties, Buildability, Pressure field, Shrinkage of concrete, Yield strength Bubble features	C. Liu et al. [7]	2022
3D-printed foam concrete	Portland cement, Silica fume, Hydroxypropyl methylcellulose, Sand, Nanomodified compound foaming agent	Yield stress, Plastic viscosity	C. Liu et al. [56]	2023

## Data Availability

No data.
